# Complete genome of a novel virulent phage ST0 lysing *Escherichia coli* H8

**DOI:** 10.1186/s40793-017-0304-9

**Published:** 2017-12-19

**Authors:** Honghui Liu, Xinchun Liu, Jinqing Li

**Affiliations:** College of Resources and Environment, University of Chinese Academy of Sciences, Beijing, 100049 China

**Keywords:** *Escherichia coli*, Virulent phage, Complete genome, Phage therapy, Antimicrobial agents

## Abstract

Phage ST0 lysing *Escherichia coli* H8 was isolated from wastewater and sequenced using an Illumina HiSeq system. Genomic analyses revealed that it was virulent phages and contained a circular double-stranded DNA genome, consisting of 170,496 nucleotides with an average G + C content of 37.67%. This study may provide possible alternative materials for phage therapy.

**Electronic supplementary material:**

The online version of this article (doi: 10.1186/s40793-017-0304-9) contains supplementary material, which is available to authorized users.

## Introduction

A large number of antibiotics were produced and widely used in medical and agricultural areas. These substances in the environment didn’t tend to be biodegradable, and were easily stored and accumulated in water and soil environment and even in the atmospheric environment [1–3]. Recently, antibiotics had been recognized as the emerging environmental pollutants, because of their potential undesirable effects on the ecosystem and human health [4–6], such as antibiotic resistance. The resistance of bacteria to current antibiotics increased the difficulty in medical treatment, which accounted for 23,000 deaths annually in the USA. The spread of antibiotic resistant bacteria and antibiotic resistance genes in the environment was a major public health issue. Obviously, strict control of the use of antibiotics and the development of a possible alternative medicine seemed extremely urgent.

Compared with antibiotics, phage therapy had the advantages of high specificity, few side effects and capacity for low-dosage use and so on [7]. In particular, it was alternative and effective to adopt phage therapy to treat diseases caused by antibiotics-resistant bacteria strains [8]. Currently phage therapy mainly had single phage treatment, multiple phage treatment and combined therapy of phage and antibiotics. More recently, bacteriophages had been intensively studied and potential application for the control of 10.1601/nm.3093 in livestock, aquaculture and food products [9–11].

In this work, phage ST0 against 10.1601/nm.3093 H8 was isolated from industrial wastewater in China. Its morphology, complete genome sequence and bioinformatics analysis were explored. This could provide a better understanding to the development of a possible alternative medicines and biocontrol agents.

## Organism information

### Classification and features

**Fig. 1:**
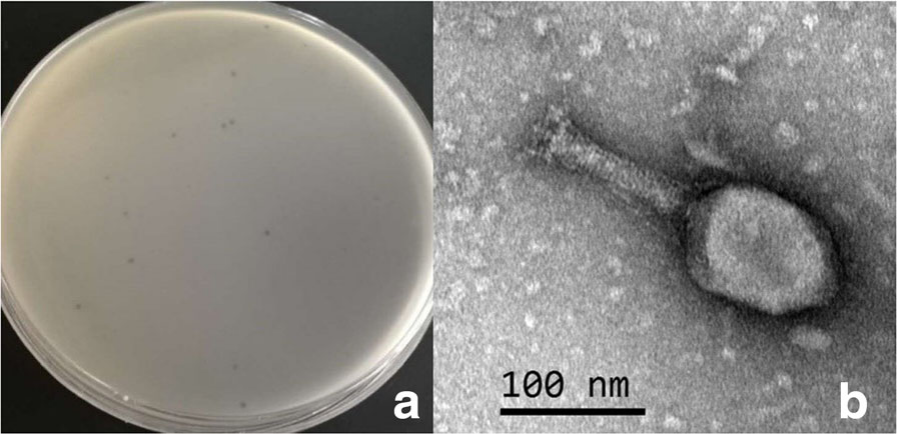
Characterization of phage ST0 morphology. **a** The negative colony; **b** TEM image

**Fig. 2:**
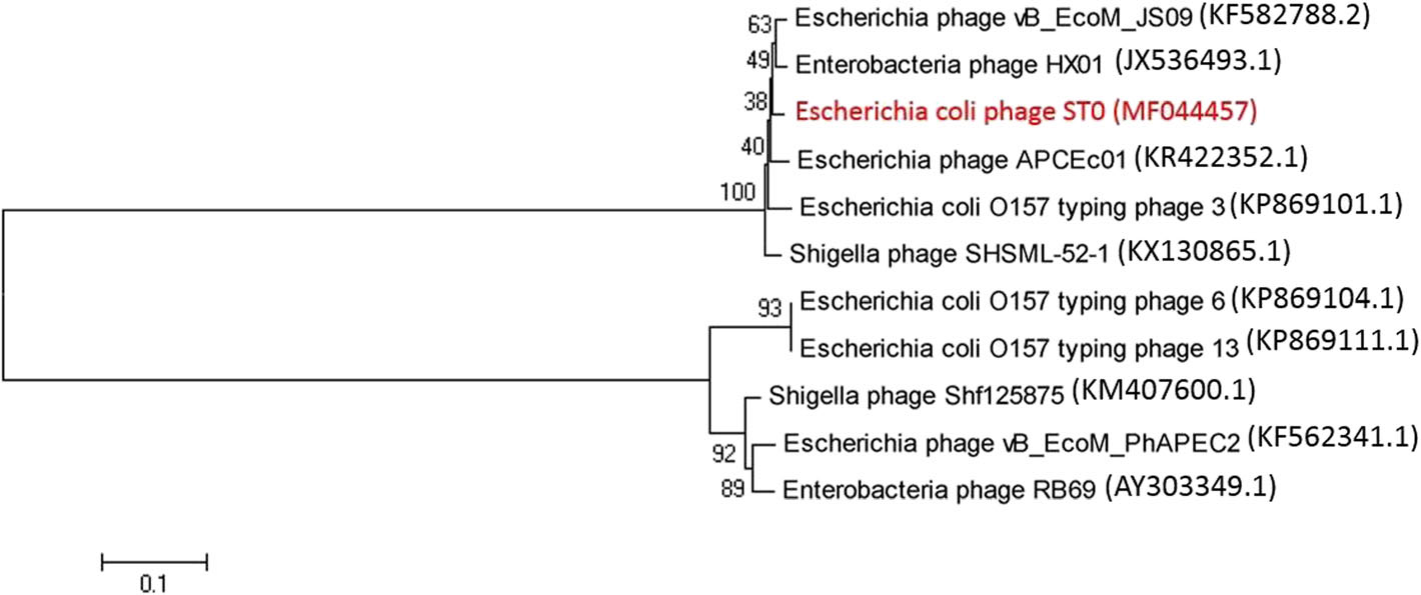
Neighbor-joining phylogenetic tree based on the complete genome sequence of phage ST0

10.1601/nm.3093 H8 (ST100), the host to isolate virulent phages, carrying shiga toxin genes (*stx1*, *stx2*) was obtained from the Chinese Center for Disease Control and Prevention. Phage ST0 was isolated from a sewage treatment plant of wastewater in Beijing. The isolation, propagation and titration of phage was done according to the methods described previously [12]. Phage ST0 generated clear plaques on double-layer plate (Fig. 1a), indicating that it was a virulent phage. The diameter of plaques was 1–2 mm. The transmission electron microscopy image (Fig. 1b) showed that phage ST0 had an icosahedral head approximately 120 nm in long diameter and 80 nm in short diameter. Its long tail was about 120 nm in length and 20 nm in diameter. Phylogenetic analysis based on complete genome sequences revealed that phage ST0 was closely related to *Enterobacteria* phage HX01 (Accession JX536493.1), whereas the score was relatively low (Fig. 2). A summary of the isolation and general phylogenetic features of phage ST0 are shown in Table 1.

**Table 1:** Classification and general features of *Genusspecies* strain designation^T^ [19]

MIGS ID	Property	Term	Evidence code^a^
	Classification	Domain: Viruses, dsDNA viruses	TAS [21]
		Phylum: unassigned	
		Class: unassigned	
		Order: *Caudovirales*	TAS [21]
		Family: *Siphoviridae*	TAS [21]
		Genus: unassigned	
		Species: unassigned	
		(Type) strain: unassigned	
	Gram stain	N/A	
	Cell shape	N/A	
	Motility	N/A	
	Sporulation	N/A	
	Temperature range	N/A	
	Optimum temperature	N/A	
	pH range; Optimum	N/A	
	Carbon source	N/A	
MIGS-6	Habitat	Water	IDA
MIGS-6.3	Salinity	N/A	
MIGS-22	Oxygen requirement	N/A	
MIGS-15	Biotic relationship	Intracellular parasite of *Escherichia coli* H8	IDA
MIGS-14	Pathogenicity	Lytic phage of *Escherichia coli* H8	IDA
MIGS-4	Geographic location	China	IDA
MIGS-5	Sample collection	April, 2017	IDA
MIGS-4.1	Latitude	40°N	IDA
MIGS-4.2	Longitude	116°E	IDA
MIGS-4.4	Altitude	Unknown	

^a^Evidence codes - *IDA* inferred from direct assay, *TAS* traceable author statement (i.e., a direct report exists in the literature). These evidence codes are from the Gene Ontology project [20]

## Genome sequencing information

### Genome project history

Phage ST0 infecting 10.1601/nm.3093 was isolated and sequenced because of its potential for use in phage therapy. The genome sequence and annotation are available in GenBank (MF044457). These data were summarized in Table 2.

**Table 2:** Project information

MIGS ID	Property	Term
MIGS 31	Finishing quality	Complete
MIGS-28	Libraries used	Illumina library
MIGS 29	Sequencing platforms	Illumina HiSeq 2500
MIGS 31.2	Fold coverage	5000-folds
MIGS 30	Assemblers	Abyss 1.3.6
MIGS 32	Gene calling method	PHASTER
	Locus Tag	N/A^a^
	Genbank ID	MF044457
	GenBank Date of Release	28-JUN-2017
	GOLD ID	N/A^a^
	BIOPROJECT	N/A^a^
MIGS 13	Source Material Identifier	N/A^a^
	Project relevance	Isolation and application of phages infecting *Escherichia coli*

^a^Not available

### Growth conditions and genomic DNA preparation

Phage ST0 was isolated from a wastewater sample that was filtered through a 0.22-μm polycarbonate membrane filter (Millipore, Bedford, MA, USA). The host strain 10.1601/nm.3093 H8 was cultured at 37 °C using LB medium [13]. Phage DNA was extracted as described by Sambrook and Russell [14]. The phage lysates were concentrated in polyethylene glycol 8000 and bacterial nucleic acids were removed from phage lysates by DNase I (Sigma-Aldrich, Oakville, Canada) and RNaseA (Sigma-Aldrich). Then the phage particles were amplified and stored in SM buffer (100 mM NaCl, 8 mM MgSO4, 50 mM Tris-HCl [pH 7.5]) at 4 °C.

### Genome sequencing and assembly

DNA was sequenced using the Illumina HiSeq 2500 platform in Beijing Fixgene Tech Co., Ltd. More than 5000-fold coverage of the phage genome is generated by sequencing the cloned fragments. The paired-end reads were assembled using the abyss v. 1.3.6. Possible tRNAs in the genome were determined using tRNAscan-SE. These data were summarized in Table 3.

**Table 3:** Genome statistics

Attribute	Value	% of Total
Genome size (bp)	170,496	100.00
DNA coding (bp)	157,610	92.44
DNA G + C (bp)	64,218	37.67
DNA scaffolds	0	100.00
Total genes	269	100.00
Protein coding genes	269	100.00
RNA genes	12	0.55
Pseudo genes	0	0.00
Genes in internal clusters	0	0.00
Genes with function prediction	89	33.09
Genes assigned to COGs	121	44.98
Genes with Pfam domains	0	0.00
Genes with signal peptides	0	0.00
Genes with transmembrane helices	0	0.00
CRISPR repeats	0	0.00

### Genome annotation

The potential ORFs were predicted using PHASTER [15]. Putative protein function of ORFs was annotated by BLASTp against NCBI database and HMMER search against the COG database [16] (These data were summarized in Table 4). The map of a circular representation of phage ST0 genome was generated using CGView Server. Neighbor joining tree was drawn by MEGA 5.05 [17].

**Table 4:** Number of genes associated with general COG functional categories

Code	Value	%age	Description
J	2	0.74	Translation, ribosomal structure and biogenesis
A	0	0.00	RNA processing and modification
K	7	2.60	Transcription
L	15	5.58	Replication, recombination and repair
B	0	0.00	Chromatin structure and dynamics
D	2	0.74	Cell cycle control, Cell division, chromosome partitioning
V	2	0.74	Defense mechanisms
T	3	1.12	Signal transduction mechanisms
M	5	1.86	Cell wall/membrane biogenesis
N	0	0.00	Cell motility
U	0	0.00	Intracellular trafficking and secretion
O	6	2.23	Posttranslational modification, protein turnover, chaperones
C	2	0.74	Energy production and conversion
G	4	1.49	Carbohydrate transport and metabolism
E	6	2.23	Amino acid transport and metabolism
F	9	3.35	Nucleotide transport and metabolism
H	2	0.74	Coenzyme transport and metabolism
I	0	0.00	Lipid transport and metabolism
P	2	0.74	Inorganic ion transport and metabolism
Q	0	0.00	Secondary metabolites biosynthesis, transport and catabolism
R	0	0.00	General function prediction only
S	54	20.07	Function unknown
–	148	55.02	Not in COGs

The total is based on the total number of protein coding genes in the genome

## Genome properties

The complete genome sequence of phage ST0 had been deposited in GenBank with the accession number MF044457. The complete genome sequence of phage ST0 consisted of 170,496 bp and was circular double-stranded DNA with an average GC content of 37.67%. There were ten tRNAs detected in this genome indicating that phage ST0 could be reliant on its tRNAs after entering into the hosts.

**Fig. 3:**
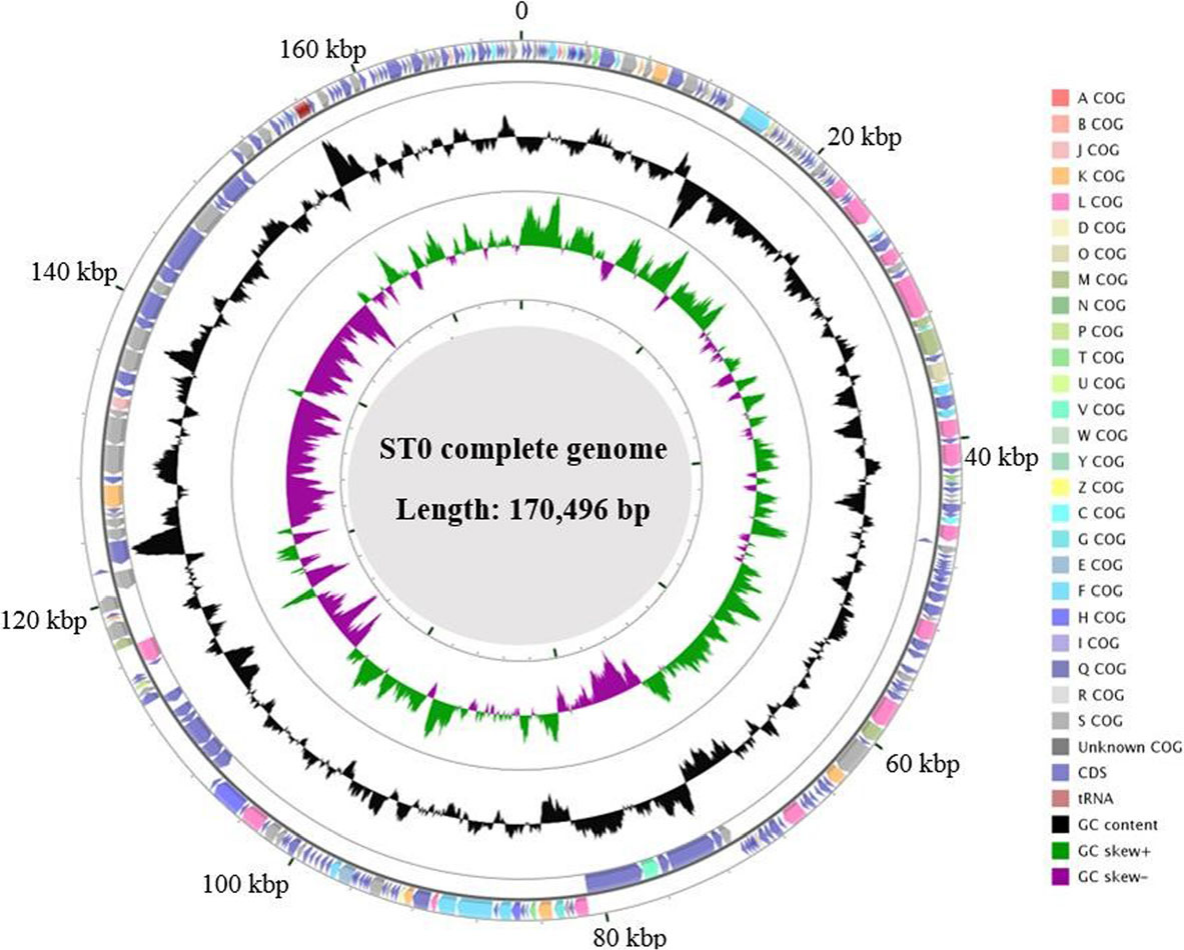
CGView Server map of phage ST0 complete genome

A total of 269 ORFs were predicted in this complete genome, compared with those from the NCBI database (Fig. 3; Additional file 1: Table S1). These ORFs showed more than 94% identity with 18 different phage strains. Of those, 41 ORFs were predicted in the minus strand and others were in the plus strand. Eighty nine putative ORFs were predicted to have unkown functions.

## Insights from the genome sequence

Phage ST0 possessed replication-related genes encoding DNA polymerase (ORF64), DNA primase/helicase (ORF76, ORF87, ORF101 and ORF195), DNA ligase (ORF179), topoisomerase (ORF110, ORF121), DNA binding protein (ORF139, ORF142), terminase (ORF211 and ORF212) and other related proteins (ORF7, ORF18, ORF33, ORF34, ORF60-62, ORF89, ORF138, ORF146-147, ORF149-151, ORF159, ORF166, ORF231 and ORF259). Terminase was observed in the phage ST0 genome, and plays the essential role in the double-stranded DNA packaging process. Terminase generally composed of two sub-units identifies the pre-capsid protein and the specific packaging sites, providing energy to packaging process through hydrolysis of ATP [17, 18]. This showed that phage ST0 possibly depended on its own terminase to obtain these kinds of function, while many phages lacked this enzyme.

Phage ST0 may be dependent on its own gene transcription and translation, because it possessed RNA-related enzymes such as tRNA synthetase modifier (ORF2), putative thioredoxin (ORF28), RNA polymerase sigma factor (ORF49), RNA ligase (ORF152 and ORF200) and other related proteins (ORF59, ORF63, ORF123, ORF140, ORF165, ORF181) to regulate gene expression.

Forty one genes that were related to the structure of phage ST0 were identified in its genome, including phage virion protein and tail tube. The ORFs related to structure were mainly concentrated between 106,926 bp and 153,860 bp, which facilitated the rapid assembly of phages and also reflected the lowest energy principle in nature.

The presence of lysozyme (ORF257) and holing (ORF132) indicated that phage ST0 was a lytic phage. Moreover, other several active factors played a quite important role in host cell lysis and inhibition of host cell growth, such as exonuclease (ORF102), inhibitor of host transcription (ORF153), nudix hydrolase (ORF256) and rIIA protein (ORF113, ORF114 and ORF169).

## Conclusions

The morphology, complete genome sequence and bioinformatics analysis showed phage ST0 was a novel virulent phage infecting and lysing 10.1601/nm.3093 H8, which may provide a better understanding to the development of a possible alternative medicines and biocontrol agents.

## Additional file

Additional file 1: Table S1. Predicted protein function of phage ST0. (DOCX 53 kb)

## Abbreviations

TEM: Transmission electron microscopy
