# Correction to: Complete genome of a novel virulent phage ST0 lysing *Escherichia coli* H8

**DOI:** 10.1186/s40793-018-0319-x

**Published:** 2018-07-04

**Authors:** Honghui Liu, Xinchun Liu, Jinqing Li

**Affiliations:** College of Resources and Environment, University of Chinese Academy of Sciences, Beijing, 100049 China

## Correction

Table 1 of this original publication contained an error. The updated Table 1 is published in this correction article [1].

**Table 1:** Classification and general features of *Genusspecies* strain designation^T^ [19]

MIGS ID	Property	Term	Evidence code^a^
	Classification	Domain: Viruses, dsDNA viruses	TAS [21]
		Phylum: unassigned	
		Class: unassigned	
		Order: *Caudovirales*	TAS [21]
		Family: *Myoviridae*	TAS [21]
		Genus: unassigned	
		Species: unassigned	
		(Type) strain: unassigned	
	Gram stain	N/A	
	Cell shape	N/A	
	Motility	N/A	
	Sporulation	N/A	
	Temperature range	N/A	
	Optimum temperature	N/A	
	pH range; Optimum	N/A	
	Carbon source	N/A	
MIGS-6	Habitat	Water	IDA
MIGS-6.3	Salinity	N/A	
MIGS-22	Oxygen requirement	N/A	
MIGS-15	Biotic relationship	Intracellular parasite of *Escherichia coli* H8	IDA
MIGS-14	Pathogenicity	Lytic phage of *Escherichia coli* H8	IDA
MIGS-4	Geographic location	China	IDA
MIGS-5	Sample collection	April, 2017	IDA
MIGS-4.1	Latitude	40°N	IDA
MIGS-4.2	Longitude	116°E	IDA
MIGS-4.4	Altitude	Unknown	

^a^Evidence codes - *IDA* inferred from direct assay, *TAS* traceable author statement (i.e., a direct report exists in the literature). These evidence codes are from the Gene Ontology project [20]
