# Supplementary material for: Complete genome of a novel virulent phage ST0 lysing Escherichia coli H8
Source: Stand Genomic Sci. 2017 Dec 19;12:85. doi: 10.1186/s40793-017-0304-9 (PMC5738172; doi:10.1186/s40793-017-0304-9)
Supplement: Additional file 1: Table S1. — Predicted protein function of phage ST0. (DOCX 53 kb) [file 40793_2017_304_MOESM1_ESM.docx]

**Table S1.** Predicted protein function of phage ST0.

| **ORF** | **Start** | **End** | **Strand** | **Predicted protein functions** | **In NCBI database** | **E-value, identity** |
| --- | --- | --- | --- | --- | --- | --- |
| 1 | 53 | 337 | + | hypothetical protein | *Enterobacteria* phage RB69 | 1e-61, 100% |
| 2 | 343 | 681 | + | valyl-tRNA synthetase modifier | [Escherichia](http://doi.org/10.1601/nm.3092) phage APCEc01 | 8e-76, 99% |
| 3 | 761 | 1141 | + | macro domain protein | [Escherichia coli](http://doi.org/10.1601/nm.3093) O157 typing phage 3 | 6e-87, 99% |
| 4 | 1138 | 1350 | + | hypothetical protein | Bacteria | 7e-45, 99% |
| 5 | 1347 | 1553 | + | hypothetical protein | [Escherichia](http://doi.org/10.1601/nm.3092) phage phiE142 | 9e-44, 100% |
| 6 | 1550 | 1732 | + | hypothetical protein | [Escherichia](http://doi.org/10.1601/nm.3092) phage vB_EcoM_JS09 | 6e-34, 100% |
| 7 | 1742 | 2323 | + | thymidine kinase | [Clostridioides difficile](http://doi.org/10.1601/nm.28959) | 7e-142, 100% |
| 8 | 2351 | 2563 | + | hypothetical protein | [Clostridioides difficile](http://doi.org/10.1601/nm.28959) | 9e-42, 99% |
| 9 | 2600 | 2878 | + | hypothetical protein | [Escherichia coli](http://doi.org/10.1601/nm.3093) O157 typing phage 3 | 2e-62, 99% |
| 10 | 2980 | 3159 | + | hypothetical protein | [Clostridioides difficile](http://doi.org/10.1601/nm.28959) | 9e-36, 100% |
| 11 | 3167 | 3334 | + | hypothetical protein | [Escherichia](http://doi.org/10.1601/nm.3092) phage vB_EcoM_JS09 | 3e-29, 93% |
| 12 | 3313 | 3426 | + | hypothetical protein | [Escherichia](http://doi.org/10.1601/nm.3092) phage vB_EcoM_JS09 | 7e-18, 97% |
| 13 | 3426 | 3641 | + | hypothetical protein | [Escherichia](http://doi.org/10.1601/nm.3092) phage vB_EcoM_PhAPEC2 | 7e-43, 99% |
| 14 | 3687 | 3809 | + | hypothetical protein | [Escherichia](http://doi.org/10.1601/nm.3092) phage HX01 | 7e-19, 100% |
| 15 | 3806 | 3985 | + | hypothetical protein | [Escherichia](http://doi.org/10.1601/nm.3092) phage vB_EcoM_JS09 | 2e-34, 98% |
| 16 | 3987 | 4517 | + | hypothetical protein | [Escherichia](http://doi.org/10.1601/nm.3092) phage vB_EcoM_JS09 | 1e-125, 98% |
| 17 | 4527 | 5000 | + | hypothetical protein | [Escherichia coli](http://doi.org/10.1601/nm.3093) O157 typing phage 3 | 1e-109, 99% |
| 18 | 5000 | 5986 | + | putative nucleotidyltransferase | [Escherichia coli](http://doi.org/10.1601/nm.3093) O157 typing phage 3 | 0.0, 99% |
| 19 | 6018 | 6299 | + | hypothetical protein | [Clostridioides difficile](http://doi.org/10.1601/nm.28959) | 4e-59, 98% |
| 20 | 6419 | 7399 | + | hypothetical protein | [Escherichia](http://doi.org/10.1601/nm.3092) phage vB_EcoM_JS09 | 0.0, 100% |
| 21 | 7538 | 7825 | + | hypothetical protein | [Escherichia](http://doi.org/10.1601/nm.3092) phage APCEc01 | 2e-162, 100% |
| 22 | 7884 | 8411 | + | hypothetical protein | [Escherichia](http://doi.org/10.1601/nm.3092) phage vB_EcoM_JS09 | 2e-122, 99% |
| 23 | 8474 | 9469 | + | hypothetical protein | [Escherichia](http://doi.org/10.1601/nm.3092) phage vB_EcoM_JS09 | 0.0, 99% |
| 24 | 9525 | 10484 | + | hypothetical protein | [Escherichia](http://doi.org/10.1601/nm.3092) phage phiE142 | 0.0, 99% |
| 25 | 10547 | 11491 | + | hypothetical protein | [Escherichia coli](http://doi.org/10.1601/nm.3093) O157 typing phage 3 | 0.0, 99% |
| 26 | 11491 | 11796 | + | hypothetical protein | [Escherichia](http://doi.org/10.1601/nm.3092) phage vB_EcoM_JS09 | 4e-69, 99% |
| 27 | 11796 | 12209 | + | hypothetical protein | *Enterobacteria* phage RB69 | 1e-95, 100% |
| 28 | 12202 | 12465 | + | putative thioredoxin | [Escherichia](http://doi.org/10.1601/nm.3092) phage vB_EcoM_PhAPEC2 | 4e-56, 95% |
| 29 | 12462 | 12758 | + | hypothetical protein | [Escherichia](http://doi.org/10.1601/nm.3092) phage APCEc01 | 2e-61, 92% |
| 30 | 12777 | 13133 | + | hypothetical protein | [Escherichia](http://doi.org/10.1601/nm.3092) phage vB_EcoM_JS09 | 3e-79, 98% |
| 31 | 13105 | 13272 | + | hypothetical protein | [Escherichia](http://doi.org/10.1601/nm.3092) phage vB_EcoM_JS09 | 1e-31, 98% |
| 32 | 13262 | 13432 | + | hypothetical protein | [Escherichia](http://doi.org/10.1601/nm.3092) phage phiE142 | 2e-32, 98% |
| 33 | 13472 | 13945 | + | recombination endonuclease VII | [Escherichia](http://doi.org/10.1601/nm.3092) phage APCEc01 | 4e-113, 99% |
| 34 | 14754 | 16571 | + | Ribonucleotide reductase of class III (anaerobic) large subunit | [Escherichia](http://doi.org/10.1601/nm.3092) phage vB_EcoM-UFV13 | 0.0, 94% |
| 35 | 16568 | 17038 | + | hypothetical protein | *Enterobacteria* phage T2H | 6e-111, 99% |
| 36 | 17031 | 17144 | + | hypothetical protein | [Shigella](http://doi.org/10.1601/nm.3329) phage SP18 | 2e-16, 95% |
| 37 | 17153 | 17368 | + | hypothetical protein | [Escherichia](http://doi.org/10.1601/nm.3092) phage HX01 | 6e-40, 99% |
| 38 | 17419 | 17688 | + | hypothetical protein | [Escherichia](http://doi.org/10.1601/nm.3092) phage vB_EcoM_JS09 | 4e-57, 97% |
| 39 | 17654 | 17977 | + | hypothetical protein | [Escherichia](http://doi.org/10.1601/nm.3092) phage vB_EcoM_PhAPEC2 | 5e-71, 99% |
| 40 | 18143 | 18325 | + | hypothetical protein | [Escherichia](http://doi.org/10.1601/nm.3092) phage vB_EcoM_JS09 | 1e-36, 100% |
| 41 | 18322 | 18570 | + | hypothetical protein | [Escherichia](http://doi.org/10.1601/nm.3092) phage APCEc01 | 9e-49, 95% |
| 42 | 18578 | 18871 | + | hypothetical protein | [Escherichia](http://doi.org/10.1601/nm.3092) phage vB_EcoM_JS09 | 1e-63, 100% |
| 43 | 18879 | 19013 | + | hypothetical protein | [Shigella](http://doi.org/10.1601/nm.3329) phage Shf125875 | 7e-24, 98% |
| 44 | 19010 | 19210 | + | hypothetical protein | [Escherichia](http://doi.org/10.1601/nm.3092) phage vB_EcoM_JS09 | 9e-42, 98% |
| 45 | 19276 | 19515 | + | hypothetical protein | [Escherichia](http://doi.org/10.1601/nm.3092) phage HX01 | 6e-50, 99% |
| 46 | 19582 | 19914 | + | hypothetical protein | [Escherichia](http://doi.org/10.1601/nm.3092) phage vB_EcoM_JS09 | 1e-73, 100% |
| 47 | 19911 | 20138 | + | hypothetical protein | [Escherichia](http://doi.org/10.1601/nm.3092) phage vB_EcoM_JS09 | 2e-44, 99% |
| 48 | 20135 | 20404 | + | hypothetical protein | [Clostridioides difficile](http://doi.org/10.1601/nm.28959) | 3e-59, 100% |
| 49 | 20477 | 21034 | + | RNA polymerase sigma factor | [Escherichia](http://doi.org/10.1601/nm.3092) phage vB_EcoM_JS09 | 3e-134, 99% |
| 50 | 21024 | 21233 | + | hypothetical protein | [Escherichia](http://doi.org/10.1601/nm.3092) phage APCEc01 | 9e-43, 100% |
| 51 | 21235 | 21558 | + | hypothetical protein | *Enterobacteria* phage RB69 | 8e-70, 100% |
| 52 | 21533 | 21739 | + | hypothetical protein | [Clostridioides difficile](http://doi.org/10.1601/nm.28959) | 1e-42, 100% |
| 53 | 21779 | 21952 | + | hypothetical protein | [Escherichia](http://doi.org/10.1601/nm.3092) phage APCEc01 | 3e-34, 100% |
| 54 | 22022 | 23041 | + | recombination endonuclease subunit | [Escherichia](http://doi.org/10.1601/nm.3092) phage vB_EcoM_JS09 | 0.0, 99% |
| 55 | 23038 | 23289 | + | hypothetical protein | [Escherichia](http://doi.org/10.1601/nm.3092) phage vB_EcoM_PhAPEC2 | 2e-54, 100% |
| 56 | 23282 | 23521 | + | hypothetical protein | [Clostridioides difficile](http://doi.org/10.1601/nm.28959) | 4e-51, 100% |
| 57 | 23518 | 25206 | + | endonuclease subunit | *Enterobacteria* phage RB69 | 0.0, 99% |
| 58 | 25997 | 26188 | + | hypothetical protein | [Clostridioides difficile](http://doi.org/10.1601/nm.28959) | 6e-38, 100% |
| 59 | 26201 | 26617 | + | RpbA RNA polymerase binding protein | *Enterobacteria* phage RB69 | 4e-99, 100% |
| 60 | 26660 | 27346 | + | sliding clamp | [Clostridioides difficile](http://doi.org/10.1601/nm.28959) | 2e-165, 100% |
| 61 | 27422 | 28384 | + | clamp loader subunit | [Shigella](http://doi.org/10.1601/nm.3329) phage Shf125875 | 0.0, 100% |
| 62 | 28386 | 28949 | + | clamp loader small subunit | *Enterobacteria* phage RB69 | 1e-134, 100% |
| 63 | 28952 | 29320 | + | translation repressor protein | *Enterobacteria* phage RB69 | 9e-85, 100% |
| 64 | 29402 | 32113 | + | DNA polymerase | [Escherichia](http://doi.org/10.1601/nm.3092) phage vB_EcoM_JS09 | 0.0, 100% |
| 65 | 32154 | 32789 | + | hypothetical protein | *Enterobacteria* phage RB69 | 3e-153, 100% |
| 66 | 32786 | 32929 | + | hypothetical protein | [Shigella](http://doi.org/10.1601/nm.3329) phage Shf125875 | 1e-23, 96% |
| 67 | 32971 | 34656 | + | hypothetical protein | [Escherichia](http://doi.org/10.1601/nm.3092) phage vB_EcoM_JS09 | 0.0, 100% |
| 68 | 34656 | 35042 | + | hypothetical protein | [Clostridioides difficile](http://doi.org/10.1601/nm.28959) | 3e-91, 100% |
| 69 | 35097 | 36257 | + | hypothetical protein | [Escherichia](http://doi.org/10.1601/nm.3092) phage vB_EcoM_JS09 | 0.0, 99% |
| 70 | 36254 | 36397 | + | hypothetical protein | [Escherichia coli](http://doi.org/10.1601/nm.3093) O157 typing phage 3 | 7e-25, 100% |
| 71 | 36536 | 37252 | + | hypothetical protein | [Escherichia](http://doi.org/10.1601/nm.3092) phage vB_EcoM_JS09 | 9e-180, 99% |
| 72 | 37252 | 38151 | + | hypothetical protein | [Escherichia](http://doi.org/10.1601/nm.3092) phage APCEc01 | 0.0, 99% |
| 73 | 38153 | 38701 | + | hypothetical protein | [Shigella](http://doi.org/10.1601/nm.3329) phage Shf125875 | 3e-132, 100% |
| 74 | 38801 | 39973 | + | hypothetical protein | *Enterobacteria* phage RB69 | 0.0, 100% |
| 75 | 39966 | 40307 | + | hypothetical protein | [Escherichia](http://doi.org/10.1601/nm.3092) phage vB_EcoM_JS09 | 3e-77, 100% |
| 76 | 40317 | 41759 | + | helicase | [Escherichia](http://doi.org/10.1601/nm.3092) phage vB_EcoM_JS09 | 0.0, 99% |
| 77 | 41848 | 42222 | + | hypothetical protein | [Escherichia](http://doi.org/10.1601/nm.3092) phage vB_EcoM_PhAPEC2 | 2e-85, 98% |
| 78 | 42278 | 42595 | + | hypothetical protein | *Enterobacteria* phage RB69 | 1e-73, 100% |
| 79 | 42592 | 42783 | + | hypothetical protein | Phage Rb69 | 3e-37, 98% |
| 80 | 42785 | 42997 | + | hypothetical protein | *Enterobacteria* phage RB69 | 1e-44, 100% |
| 81 | 43058 | 43426 | + | hypothetical protein | [Escherichia](http://doi.org/10.1601/nm.3092) phage vB_EcoM_JS09 | 1e-84, 97% |
| 82 | 43488 | 43736 | + | hypothetical protein | *Enterobacteria* phage RB69 | 2e-49, 100% |
| 83 | 43800 | 44093 | + | hypothetical protein | [Clostridioides difficile](http://doi.org/10.1601/nm.28959) | 2e-66, 100% |
| 84 | 44095 | 44745 | + | hypothetical protein | [Escherichia coli](http://doi.org/10.1601/nm.3093) O157 typing phage 3 | 1e-156, 99% |
| 85 | 44747 | 44944 | + | hypothetical protein | *Enterobacteria* phage RB69 | 1e-38, 98% |
| 86 | 44964 | 45431 | + | hypothetical protein | [Escherichia](http://doi.org/10.1601/nm.3092) phage vB_EcoM_JS09 | 2e-109, 99% |
| 87 | 45471 | 46493 | + | DNA primase/helicase | [Shigella](http://doi.org/10.1601/nm.3329) phage SHSML-52-1 | 0.0, 99% |
| 88 | 46687 | 46490 | - | hypothetical protein | [Escherichia](http://doi.org/10.1601/nm.3092) phage HX01 | 6e-35, 97% |
| 89 | 46776 | 47297 | + | dCTP pyrophosphatase | [Clostridioides difficile](http://doi.org/10.1601/nm.28959) | 3e-126, 100% |
| 90 | 47343 | 47579 | + | capsid and scaffold protein | [Shigella](http://doi.org/10.1601/nm.3329) phage SHSML-52-1 | 5e-50, 99% |
| 91 | 47683 | 47910 | + | hypothetical protein | [Escherichia](http://doi.org/10.1601/nm.3092) phage vB_EcoM_JS09 | 6e-49, 100% |
| 92 | 47876 | 48124 | + | hypothetical protein | [Escherichia](http://doi.org/10.1601/nm.3092) phage APCEc01 | 9e-52, 98% |
| 93 | 48121 | 48300 | + | hypothetical protein | [Escherichia](http://doi.org/10.1601/nm.3092) phage vB_EcoM_JS09 | 5e-34, 98% |
| 94 | 48300 | 48764 | + | hypothetical protein | [Escherichia coli](http://doi.org/10.1601/nm.3093) O157 typing phage 3 | 4e-109, 99% |
| 95 | 48781 | 48945 | + | hypothetical protein | [Clostridioides difficile](http://doi.org/10.1601/nm.28959) | 4e-31, 100% |
| 96 | 48942 | 49106 | + | hypothetical protein | [Escherichia](http://doi.org/10.1601/nm.3092) phage vB_EcoM_PhAPEC2 | 3e-28, 98% |
| 97 | 49162 | 49743 | + | hypothetical protein | [Escherichia coli](http://doi.org/10.1601/nm.3093) O157 typing phage 3 | 9e-141, 98% |
| 98 | 49801 | 50409 | + | hypothetical protein | [Escherichia coli](http://doi.org/10.1601/nm.3093) O157 typing phage 3 | 1e-145, 97% |
| 99 | 50562 | 51308 | + | hypothetical protein | *Enterobacteria* phage RB69 | 3e-179, 99% |
| 100 | 51311 | 51622 | + | hypothetical protein | [Escherichia](http://doi.org/10.1601/nm.3092) phage vB_EcoM_PhAPEC2 | 5e-68, 98% |
| 101 | 51619 | 52932 | + | DNA helicase | [Escherichia](http://doi.org/10.1601/nm.3092) phage APCEc01 | 0.0, 99% |
| 102 | 52944 | 53621 | + | exonuclease A | [Shigella](http://doi.org/10.1601/nm.3329) phage SHSML-52-1 | 1e-166, 100% |
| 103 | 53687 | 54148 | + | hypothetical protein | [Escherichia](http://doi.org/10.1601/nm.3092) phage vB_EcoM_JS09 | 2e-166, 98% |
| 104 | 54784 | 55203 | + | hypothetical protein | [Escherichia](http://doi.org/10.1601/nm.3092) phage vB_EcoM_JS09 | 6e-94, 96% |
| 105 | 55263 | 55787 | + | hypothetical protein | [Shigella](http://doi.org/10.1601/nm.3329) phage Shf125875 | 5e-127, 99% |
| 106 | 55845 | 56072 | + | hypothetical protein | [Escherichia](http://doi.org/10.1601/nm.3092) phage HX01 | 2e-47, 100% |
| 107 | 56072 | 56482 | + | hypothetical protein | [Escherichia coli](http://doi.org/10.1601/nm.3093) O157 typing phage 3 | 5e-95, 98% |
| 108 | 56485 | 56664 | + | hypothetical protein | [Escherichia](http://doi.org/10.1601/nm.3092) phage vB_EcoM_JS09 | 1e-36, 100% |
| 109 | 56667 | 57092 | + | hypothetical protein | [Escherichia](http://doi.org/10.1601/nm.3092) phage phiE142 | 3e-95, 99% |
| 110 | 57156 | 58973 | + | topoisomerase IV subunit B | [Shigella](http://doi.org/10.1601/nm.3329) phage SHSML-52-1 | 0.0, 99% |
| 111 | 59016 | 60116 | + | hypothetical protein | [Escherichia](http://doi.org/10.1601/nm.3092) phage vB_EcoM_PhAPEC2 | 0.0, 99% |
| 112 | 60209 | 60409 | + | hypothetical protein | [Escherichia](http://doi.org/10.1601/nm.3092) phage HX01 | 5e-37, 98% |
| 113 | 60422 | 62635 | + | rIIA protein | [Shigella](http://doi.org/10.1601/nm.3329) phage Shf125875 | 0.0, 99% |
| 114 | 62645 | 63592 | + | rIIB protector from prophage-induced early lysis | [Escherichia](http://doi.org/10.1601/nm.3092) phage vB_EcoM_JS09 | 0.0, 99% |
| 115 | 63635 | 63922 | + | hypothetical protein | [Escherichia](http://doi.org/10.1601/nm.3092) phage vB_EcoM_JS09 | 5e-63, 100% |
| 116 | 63939 | 64415 | + | hypothetical protein | [Escherichia](http://doi.org/10.1601/nm.3092) phage vB_EcoM_JS09 | 2e-112, 99% |
| 117 | 64484 | 64747 | + | hypothetical protein | *Enterobacteria* phage RB69 | 1e-57, 100% |
| 118 | 64998 | 65198 | + | hypothetical protein | [Escherichia](http://doi.org/10.1601/nm.3092) phage vB_EcoM_PhAPEC2 | 2e-41, 100% |
| 119 | 65275 | 65721 | + | hypothetical protein | [Shigella](http://doi.org/10.1601/nm.3329) phage Shf125875 | 3e-104, 99% |
| 120 | 65774 | 65923 | + | hypothetical protein | [Escherichia](http://doi.org/10.1601/nm.3092) phage HX01 | 4e-25, 100% |
| 121 | 66065 | 67390 | + | DNA topisomerase II medium subunit | *Enterobacteria* phage RB69 | 0.0, 99% |
| 122 | 67576 | 67791 | + | hypothetical protein | [Escherichia](http://doi.org/10.1601/nm.3092) phage vB_EcoM_JS09 | 4e-42, 100% |
| 123 | 67895 | 68527 | + | activator of middle period transcription | [Escherichia](http://doi.org/10.1601/nm.3092) phage APCEc01 | 1e-47, 100% |
| 124 | 68538 | 68882 | + | hypothetical protein | [Escherichia](http://doi.org/10.1601/nm.3092) phage HX01 | 1e-78, 100% |
| 125 | 68879 | 69340 | + | hypothetical protein | [Escherichia coli](http://doi.org/10.1601/nm.3093) O157 typing phage 3 | 3e-110, 99% |
| 126 | 69340 | 69621 | + | hypothetical protein | [Escherichia](http://doi.org/10.1601/nm.3092) phage HX01 | 2e-61, 99% |
| 127 | 69605 | 69823 | + | hypothetical protein | *Enterobacteria* phage RB69 | 3e-44, 97% |
| 128 | 69798 | 69917 | + | hypothetical protein | [Escherichia](http://doi.org/10.1601/nm.3092) phage vB_EcoM_JS09 | 3e-18, 100% |
| 129 | 69907 | 70206 | + | hypothetical protein | [Escherichia](http://doi.org/10.1601/nm.3092) phage vB_EcoM_PhAPEC2 | 6e-63, 99% |
| 130 | 70196 | 70357 | + | hypothetical protein | [Escherichia](http://doi.org/10.1601/nm.3092) phage vB_EcoM_JS09 | 3e-28, 98% |
| 131 | 70404 | 70676 | + | hypothetical protein | [Escherichia](http://doi.org/10.1601/nm.3092) phage HX01 | 1e-56, 100% |
| 132 | 71336 | 70677 | - | holin | [Escherichia](http://doi.org/10.1601/nm.3092) phage APCEc01 | 1e-159, 99% |
| 133 | 71897 | 71346 | - | tail fibers | [Escherichia](http://doi.org/10.1601/nm.3092) phage vB_EcoM_JS09 | 1e-128, 98% |
| 134 | 75199 | 71927 | - | tail collar domain protein | [Escherichia coli](http://doi.org/10.1601/nm.3093) O157 typing phage 3 | 0.0, 88% |
| 135 | 75873 | 75208 | - | hypothetical protein | [Escherichia](http://doi.org/10.1601/nm.3092) phage vB_EcoM_JS09 | 8e-160, 100% |
| 136 | 77063 | 75936 | - | tail connector protein | [Shigella](http://doi.org/10.1601/nm.3329) phage SHSML-52-1 | 0.0, 98% |
| 137 | 80947 | 77072 | - | long tail fiber proximal subunit | [Shigella](http://doi.org/10.1601/nm.3329) phage SHSML-52-1 | 0.0, 96% |
| 138 | 81051 | 81968 | + | RnaseH | [Escherichia](http://doi.org/10.1601/nm.3092) phage vB_EcoM_JS09 | 0.0, 99% |
| 139 | 81976 | 82245 | + | double-stranded DNA binding protein | *Enterobacteria* phage RB69 | 9e-57, 100% |
| 140 | 82223 | 82561 | + | late promoter transcription accessory protein | [Escherichia](http://doi.org/10.1601/nm.3092) phage vB_EcoM_PhAPEC2 | 9e-75, 99% |
| 141 | 82558 | 83211 | + | hypothetical protein | [Escherichia coli](http://doi.org/10.1601/nm.3093) O157 typing phage 3 | 1e-156, 99% |
| 142 | 83334 | 84236 | + | single-stranded DNA binding protein | [Escherichia](http://doi.org/10.1601/nm.3092) phage vB_EcoM_PhAPEC2 | 0.0, 100% |
| 143 | 84350 | 84754 | + | hypothetical protein | [Escherichia](http://doi.org/10.1601/nm.3092) phage HX01 | 5e-91, 99% |
| 144 | 84819 | 85067 | + | hypothetical protein | [Escherichia](http://doi.org/10.1601/nm.3092) phage HX01 | 7e-54, 100% |
| 145 | 85070 | 85318 | + | hypothetical protein | [Escherichia](http://doi.org/10.1601/nm.3092) phage vB_EcoM_JS09 | 8e-54, 100% |
| 146 | 85311 | 85898 | + | dihydrofolate reductase | [Escherichia](http://doi.org/10.1601/nm.3092) phage vB_EcoM_JS09 | 8e-140, 99% |
| 147 | 85895 | 86755 | + | thymidylate synthase | [Escherichia coli](http://doi.org/10.1601/nm.3093) O157 typing phage 3 | 0.0, 98% |
| 148 | 86757 | 87011 | + | hypothetical protein | [Escherichia coli](http://doi.org/10.1601/nm.3093) O157 typing phage 3 | 3e-52, 95% |
| 149 | 87099 | 89354 | + | ribonucleoside-diphosphate reductase subunit alpha | [Escherichia](http://doi.org/10.1601/nm.3092) phage vB_EcoM_JS09 | 0.0, 99% |
| 150 | 89408 | 90586 | + | aerobic ribonucleoside diphosphate reductase small subunit | *Enterobacteria* phage RB27 | 0.0, 92% |
| 151 | 90613 | 91023 | + | endonuclease II | *Enterobacteria* phage RB69 | 1e-95, 100% |
| 152 | 91080 | 92204 | + | RNA ligase A | [Escherichia](http://doi.org/10.1601/nm.3092) phage vB_EcoM_JS09 | 0.0, 100% |
| 153 | 92204 | 92767 | + | inhibitor of host transcription | [Shigella](http://doi.org/10.1601/nm.3329) phage Shf125875 | 1e-133, 96% |
| 154 | 92755 | 93111 | + | hypothetical protein | [Escherichia](http://doi.org/10.1601/nm.3092) phage vB_EcoM_JS09 | 1e-76, 100% |
| 155 | 93108 | 93398 | + | hypothetical protein | [Escherichia](http://doi.org/10.1601/nm.3092) phage vB_EcoM_JS09 | 5e-65, 99% |
| 156 | 93395 | 93613 | + | hypothetical protein | [Shigella](http://doi.org/10.1601/nm.3329) phage Shf125875 | 8e-44, 97% |
| 157 | 93671 | 93970 | + | hypothetical protein | [Escherichia](http://doi.org/10.1601/nm.3092) phage vB_EcoM_PhAPEC2 | 1e-62, 96% |
| 158 | 93970 | 94161 | + | hypothetical protein | *Enterobacteria* phage RB69 | 2e-38, 100% |
| 159 | 94158 | 95057 | + | putative polynucleotide 5'-kinase and 3'-phosphatase | [Escherichia](http://doi.org/10.1601/nm.3092) phage vB_EcoM_PhAPEC2 | 0.0, 99% |
| 160 | 95057 | 95248 | + | hypothetical protein | [Escherichia](http://doi.org/10.1601/nm.3092) phage HX01 | 4e-37, 98% |
| 161 | 95238 | 95453 | + | hypothetical protein | *Enterobacteria* phage RB69 | 5e-44, 100% |
| 162 | 95461 | 95736 | + | hypothetical protein | [Escherichia](http://doi.org/10.1601/nm.3092) phage vB_EcoM_PhAPEC2 | 4e-59, 100% |
| 163 | 95798 | 96034 | + | hypothetical protein | *Enterobacteria* phage RB69 | 9e-47, 96% |
| 164 | 96021 | 96146 | + | hypothetical protein | [Escherichia coli](http://doi.org/10.1601/nm.3093) O157 typing phage 3 | 3e-18, 95% |
| 165 | 96155 | 97147 | + | phospho-2-dehydro-3-deoxyheptonate aldolase | [Escherichia](http://doi.org/10.1601/nm.3092) phage vB_EcoM_JS09 | 0.0, 99% |
| 166 | 97147 | 97728 | + | dCMP deaminase | [Shigella](http://doi.org/10.1601/nm.3329) phage SHSML-52-1 | 5e-141, 99% |
| 167 | 97730 | 98026 | + | hypothetical protein | [Escherichia](http://doi.org/10.1601/nm.3092) phage HX01 | 7e-66, 100% |
| 168 | 98084 | 98416 | + | gp31 head assembly cochaperone with GroEL | *Enterobacteria* phage RB69 | 4e-72, 100% |
| 169 | 98541 | 98789 | + | rIII lysis inhibition accessory protein | [Escherichia](http://doi.org/10.1601/nm.3092) phage vB_EcoM_JS09 | 2e-52, 100% |
| 170 | 98953 | 99132 | + | hypothetical protein | *Enterobacteria* phage RB69 | 3e-32, 100% |
| 171 | 99263 | 99631 | + | hypothetical protein | [Escherichia](http://doi.org/10.1601/nm.3092) phage vB_EcoM_PhAPEC2 | 2e-85, 100% |
| 172 | 99706 | 100071 | + | hypothetical protein | *Enterobacteria* phage RB69 | 1e-85, 99% |
| 173 | 100107 | 100721 | + | hypothetical protein | [Escherichia](http://doi.org/10.1601/nm.3092) phage APCEc01 | 3e-148, 98% |
| 174 | 100778 | 100975 | + | hypothetical protein | [Escherichia](http://doi.org/10.1601/nm.3092) phage vB_EcoM_JS09 | 2e-39, 100% |
| 175 | 100965 | 101177 | + | hypothetical protein | [Escherichia](http://doi.org/10.1601/nm.3092) phage HX01 | 5e-44, 99% |
| 176 | 101170 | 101628 | + | hypothetical protein | [Escherichia coli](http://doi.org/10.1601/nm.3093) O157 typing phage 3 | 3e-109, 100% |
| 177 | 101625 | 102440 | + | hypothetical protein | [Escherichia coli](http://doi.org/10.1601/nm.3093) O157 typing phage 3 | 0.0, 99% |
| 178 | 102450 | 102719 | + | hypothetical protein | [Escherichia](http://doi.org/10.1601/nm.3092) phage HX01 | 2e-59, 99% |
| 179 | 102716 | 104209 | + | DNA ligase | [Escherichia](http://doi.org/10.1601/nm.3092) phage APCEc01 | 0.0, 100% |
| 180 | 104209 | 104397 | + | hypothetical protein | [Shigella](http://doi.org/10.1601/nm.3329) phage SHSML-52-1 | 3e-37, 97% |
| 181 | 104454 | 106541 | + | RNA polymerase-ADP-ribosyltransferase Alt | [Shigella](http://doi.org/10.1601/nm.3329) phage SHSML-52-1 | 0.0, 99% |
| 182 | 106600 | 106893 | + | hypothetical protein | [Escherichia](http://doi.org/10.1601/nm.3092) phage vB_EcoM_JS09 | 2e-64, 100% |
| 183 | 107888 | 106926 | - | baseplate tail tube initiator | [Escherichia](http://doi.org/10.1601/nm.3092) phage vB_EcoM_PhAPEC2 | 0.0, 99% |
| 184 | 108997 | 107888 | - | baseplate tail tube junction | [Shigella](http://doi.org/10.1601/nm.3329) phage Shf125875 | 0.0, 99% |
| 185 | 110781 | 109009 | - | baseplate hub subunit, tail length determinator | [Escherichia](http://doi.org/10.1601/nm.3092) phage vB_EcoM_PhAPEC2 | 0.0, 99% |
| 186 | 111248 | 110778 | - | baseplate hub distal subunit | [Shigella](http://doi.org/10.1601/nm.3329) phage Shf125875 | 3e-111, 100% |
| 187 | 112029 | 111259 | - | baseplate hub subunit | [Escherichia](http://doi.org/10.1601/nm.3092) phage vB_EcoM_JS09 | 0.0, 99% |
| 188 | 113180 | 112428 | - | baseplate hub assembly protein | *Enterobacteria* phage RB69 | 0.0, 100% |
| 189 | 113228 | 113854 | + | baseplate hub subunit | [Escherichia](http://doi.org/10.1601/nm.3092) phage vB_EcoM_JS09 | 4e-154, 100% |
| 190 | 113854 | 114252 | + | baseplate wedge subunit | *Enterobacteria* phage RB69 | 6e-91, 100% |
| 191 | 114333 | 114746 | + | hypothetical protein | [Escherichia](http://doi.org/10.1601/nm.3092) phage APCEc01 | 2e-94, 99% |
| 192 | 114746 | 114970 | + | hypothetical protein | [Escherichia](http://doi.org/10.1601/nm.3092) phage vB_EcoM_PhAPEC2 | 1e-46, 99% |
| 193 | 115003 | 115170 | + | hypothetical protein | [Escherichia](http://doi.org/10.1601/nm.3092) phage vB_EcoM_JS09 | 2e-32, 100% |
| 194 | 115462 | 115229 | - | hypothetical protein | [Escherichia](http://doi.org/10.1601/nm.3092) phage HX01 | 5e-46, 100% |
| 195 | 117002 | 115488 | - | helicase | [Escherichia](http://doi.org/10.1601/nm.3092) phage vB_EcoM_JS09 | 0.0, 99% |
| 196 | 117053 | 117721 | + | inhibitor of prohead protease | [Shigella](http://doi.org/10.1601/nm.3329) phage SHSML-52-1 | 5e-158, 99% |
| 197 | 117731 | 118861 | + | large head outer capsid protein | *Enterobacteria* phage RB3 | 0.0, 92% |
| 198 | 118962 | 119156 | + | hypothetical protein | *Enterobacteria* phage RB69 | 4e-37, 98% |
| 199 | 119153 | 119404 | + | hypothetical protein | [Escherichia](http://doi.org/10.1601/nm.3092) phage vB_EcoM_PhAPEC2 | 4e-55, 995 |
| 200 | 119524 | 120522 | + | RNA ligase | [Escherichia](http://doi.org/10.1601/nm.3092) phage vB_EcoM_PhAPEC2 | 0.0, 99% |
| 201 | 121834 | 120551 | - | capsid vertex protein | [Escherichia](http://doi.org/10.1601/nm.3092) phage vB_EcoM_JS09 | 0.0, 99% |
| 202 | 121936 | 122205 | + | hypothetical protein | [Escherichia](http://doi.org/10.1601/nm.3092) phage vB_EcoM_PhAPEC2 | 2e-58, 100% |
| 203 | 123826 | 122258 | - | major capsid protein | [Escherichia](http://doi.org/10.1601/nm.3092) phage vB_EcoM_PhAPEC2 | 0.0, 99% |
| 204 | 124656 | 123844 | - | prohead core protein | [Escherichia](http://doi.org/10.1601/nm.3092) phage vB_EcoM_JS09 | 0.0, 99% |
| 205 | 125331 | 124690 | - | prohead core scaffolding protein and protease | *Enterobacteria* phage RB69 | 4e-153, 100% |
| 206 | 125756 | 125331 | - | prohead core protein | *Enterobacteria* phage RB69 | 1e-94, 99% |
| 207 | 125986 | 125756 | - | hypothetical protein | [Escherichia coli](http://doi.org/10.1601/nm.3093) O157 typing phage 3 | 8e-43, 100% |
| 208 | 127557 | 125986 | - | portal vertex protein | [Escherichia](http://doi.org/10.1601/nm.3092) phage vB_EcoM_JS09 | 0.0, 100% |
| 209 | 128133 | 127642 | - | tail tube protein | [Escherichia](http://doi.org/10.1601/nm.3092) phage vB_EcoM_JS09 | 2e-116, 99% |
| 210 | 130228 | 128246 | - | tail sheath protein | [Escherichia](http://doi.org/10.1601/nm.3092) phage vB_EcoM_JS09 | 0.0, 100% |
| 211 | 132094 | 130259 | - | terminase large subunit | [Shigella](http://doi.org/10.1601/nm.3329) phage Shf125875 | 0.0, 99% |
| 212 | 132572 | 132078 | - | terminase small subunit | [Shigella](http://doi.org/10.1601/nm.3329) phage SHSML-52-1 | 6e-116, 99% |
| 213 | 133406 | 132582 | - | tail sheath stabilizer and completion protein | [Escherichia](http://doi.org/10.1601/nm.3092) phage vB_EcoM_PhAPEC2 | 0.0, 99% |
| 214 | 134046 | 133456 | - | head completion, neck hetero-dimeric protein | [Shigella](http://doi.org/10.1601/nm.3329) phage SHSML-52-1 | 3e-146, 100% |
| 215 | 135148 | 134222 | - | head completion, neck hetero-dimeric protein | [Shigella](http://doi.org/10.1601/nm.3329) phage SHSML-52-1 | 0.0, 99% |
| 216 | 136629 | 135181 | - | fibritin | [Escherichia](http://doi.org/10.1601/nm.3092) phage vB_EcoM_JS09 | 0.0, 98% |
| 217 | 138212 | 136629 | - | short tail fiber protein | [Escherichia coli](http://doi.org/10.1601/nm.3093) O157 typing phage 3 | 0.0, 99% |
| 218 | 138868 | 138209 | - | baseplate wedge subunit and tail pin | [Escherichia](http://doi.org/10.1601/nm.3092) phage vB_EcoM_JS09 | 3e-157, 99% |
| 219 | 140673 | 138868 | - | hypothetical protein | [Escherichia coli](http://doi.org/10.1601/nm.3093) O157 typing phage 3 | 0.0, 99% |
| 220 | 141545 | 140673 | - | baseplate wedge tail fiber connector | [Escherichia](http://doi.org/10.1601/nm.3092) phage vB_EcoM_JS09 | 0.0, 99% |
| 221 | 142622 | 141618 | - | baseplate wedge subunit | [Escherichia](http://doi.org/10.1601/nm.3092) phage vB_EcoM_JS09 | 0.0, 100% |
| 222 | 145668 | 142615 | - | hypothetical protein | [Escherichia coli](http://doi.org/10.1601/nm.3093) O157 typing phage 3 | 0.0, 99% |
| 223 | 147683 | 145710 | - | hypothetical protein | [Escherichia coli](http://doi.org/10.1601/nm.3093) O157 typing phage 3 | 0.0, 99% |
| 224 | 147985 | 147692 | - | baseplate hub needle | [Shigella](http://doi.org/10.1601/nm.3329) phage Shf125875 | 3e-64, 99% |
| 225 | 148461 | 147988 | - | hypothetical protein | [Escherichia coli](http://doi.org/10.1601/nm.3093) O157 typing phage 3 | 4e-112, 100% |
| 226 | 150186 | 148507 | - | baseplate hub subunit and tail lysozyme | [Escherichia](http://doi.org/10.1601/nm.3092) phage APCEc01 | 0.0, 99% |
| 227 | 150815 | 150240 | - | baseplate wedge subunit | [Escherichia](http://doi.org/10.1601/nm.3092) phage vB_EcoM_JS09 | 7e-138, 99% |
| 228 | 150877 | 151326 | + | head completion protein | [Shigella](http://doi.org/10.1601/nm.3329) phage Shf125875 | 2e-106, 100% |
| 229 | 151329 | 152150 | + | hypothetical protein | [Shigella](http://doi.org/10.1601/nm.3329) phage Shf125875 | 0.0, 100% |
| 230 | 152253 | 152837 | + | tail completion and sheath stabilizer protein | [Escherichia](http://doi.org/10.1601/nm.3092) phage vB_EcoM_JS09 | 2e-143, 100% |
| 231 | 152891 | 153625 | + | deoxynucleoside monophosphate kinase | [Escherichia](http://doi.org/10.1601/nm.3092) phage APCEc01 | 1e-178, 100% |
| 232 | 153630 | 153860 | + | chaperone for tail fiber formation | [Escherichia](http://doi.org/10.1601/nm.3092) phage APCEc01 | 2e-43, 100% |
| 233 | 153860 | 154315 | + | hypothetical protein | [Escherichia](http://doi.org/10.1601/nm.3092) phage vB_EcoM_JS09 | 2e-108, 100% |
| 234 | 154393 | 154656 | + | hypothetical protein | [Escherichia coli](http://doi.org/10.1601/nm.3093) O157 typing phage 3 | 2e-54, 97% |
| 235 | 154726 | 154911 | + | hypothetical protein | [Escherichia](http://doi.org/10.1601/nm.3092) phage WG01 | 9e-34, 100% |
| 236 | 154913 | 155293 | + | hypothetical protein | [Shigella](http://doi.org/10.1601/nm.3329) phage SHBML-50-1 | 2e-85, 97% |
| 237 | 155296 | 155583 | + | hypothetical protein | [Escherichia](http://doi.org/10.1601/nm.3092) phage wV7 | 1e-62, 98% |
| 238 | 156530 | 156874 | + | hypothetical protein | [Shigella](http://doi.org/10.1601/nm.3329) phage Shf125875 | 2e-77, 96% |
| 239 | 157259 | 157885 | + | hypothetical protein | [Escherichia](http://doi.org/10.1601/nm.3092) phage vB_EcoM_JS09 | 1e-148, 99% |
| 240 | 157996 | 158301 | + | hypothetical protein | *Enterobacteria* phage RB69 | 2e-66, 97% |
| 241 | 158370 | 158534 | + | hypothetical protein | *Enterobacteria* phage RB69 | 2e-31, 100% |
| 242 | 158581 | 158808 | + | hypothetical protein | [Escherichia](http://doi.org/10.1601/nm.3092) phage vB_EcoM_PhAPEC2 | 4e-48, 99% |
| 243 | 158879 | 159472 | + | hypothetical protein | *Enterobacteria* phage RB69 | 6e-135, 99% |
| 244 | 159522 | 160118 | + | hypothetical protein | [Escherichia](http://doi.org/10.1601/nm.3092) phage vB_EcoM_JS09 | 2e-142, 97% |
| 245 | 160108 | 160482 | + | hypothetical protein | [Escherichia](http://doi.org/10.1601/nm.3092) phage vB_EcoM_PhAPEC2 | 1e-85, 98% |
| 246 | 160817 | 161122 | + | hypothetical protein | [Escherichia](http://doi.org/10.1601/nm.3092) phage vB_EcoM_JS09 | 5e-67, 99% |
| 247 | 161132 | 161362 | + | hypothetical protein | [Escherichia](http://doi.org/10.1601/nm.3092) phage vB_EcoM_JS09 | 3e-15, 80% |
| 248 | 161414 | 161613 | + | hypothetical protein | *Enterobacteria* phage RB69 | 1e-38, 100% |
| 249 | 161676 | 161915 | + | hypothetical protein | [Escherichia](http://doi.org/10.1601/nm.3092) phage phiE142 | 2e-51, 99% |
| 250 | 161944 | 162900 | + | hypothetical protein | [Escherichia coli](http://doi.org/10.1601/nm.3093) O157 typing phage 3 | 0.0, 94% |
| 251 | 162980 | 163180 | + | hypothetical protein | [Escherichia coli](http://doi.org/10.1601/nm.3093) O157 typing phage 3 | 5e-42, 100% |
| 252 | 163239 | 163544 | + | hypothetical protein | [Escherichia](http://doi.org/10.1601/nm.3092) phage Av-05 | 1e-66, 97% |
| 253 | 163546 | 164232 | + | hypothetical protein | [Escherichia](http://doi.org/10.1601/nm.3092) phage APCEc01 | 1e-168, 99% |
| 254 | 164232 | 164723 | + | hypothetical protein | [Shigella](http://doi.org/10.1601/nm.3329) phage Shf125875 | 8e-111, 97% |
| 255 | 164720 | 164956 | + | hypothetical protein | [Shigella](http://doi.org/10.1601/nm.3329) phage Shf125875 | 9e-48, 100% |
| 256 | 164946 | 165404 | + | NudE nudix hydrolase | *Enterobacteria* phage RB69 | 3e-110, 99% |
| 257 | 165439 | 165927 | + | lysozyme | [Shigella](http://doi.org/10.1601/nm.3329) phage SHSML-52-1 | 1e-115, 99% |
| 258 | 165924 | 166205 | + | hypothetical protein | [Escherichia](http://doi.org/10.1601/nm.3092) phage ime09 | 5e-58, 99% |
| 259 | 166264 | 166677 | + | endonuclease V, N-glycosylase UV repair enzyme | [Escherichia](http://doi.org/10.1601/nm.3092) phage vB_EcoM_PhAPEC2 | 4e-97, 99% |
| 260 | 166690 | 166944 | + | hypothetical protein | *Enterobacteria* phage RB69 | 6e-51, 98% |
| 261 | 167008 | 167322 | + | hypothetical protein | [Escherichia](http://doi.org/10.1601/nm.3092) phage vB_EcoM_JS09 | 6e-71, 100% |
| 262 | 167349 | 167651 | + | hypothetical protein | [Klebsiella](http://doi.org/10.1601/nm.3202) phage PKO111 | 8e-64, 98% |
| 263 | 167765 | 168304 | + | hypothetical protein | [Escherichia](http://doi.org/10.1601/nm.3092) phage vB_EcoM_PhAPEC2 | 3e-128, 96% |
| 264 | 168301 | 168609 | + | hypothetical protein | [Escherichia](http://doi.org/10.1601/nm.3092) phage vB_EcoM_PhAPEC2 | 7e-70, 98% |
| 265 | 168616 | 168978 | + | hypothetical protein | [Escherichia](http://doi.org/10.1601/nm.3092) phage vB_EcoM_PhAPEC2 | 6e-83, 100% |
| 266 | 168978 | 169202 | + | hypothetical protein | [Escherichia](http://doi.org/10.1601/nm.3092) phage vB_EcoM_JS09 | 1e-47, 99% |
| 267 | 169192 | 169458 | + | hypothetical protein | *Enterobacteria* phage RB69 | 2e-57, 99% |
| 268 | 169458 | 169757 | + | hypothetical protein | [Shigella](http://doi.org/10.1601/nm.3329) phage Shf125875 | 1e-67, 100% |
| 269 | 169814 | 170272 | + | hypothetical protein | [Escherichia](http://doi.org/10.1601/nm.3092) phage APCEc01 | 7e-109, 100% |
